# The experience of body image concerns in patients with persecutory delusions: ‘People don't want to sit next to me’

**DOI:** 10.1111/papt.12246

**Published:** 2019-08-10

**Authors:** Emily Marshall, Daniel Freeman, Felicity Waite

**Affiliations:** ^1^ Oxford Institute of Clinical Psychology Training University of Oxford UK; ^2^ Department of Psychiatry University of Oxford UK; ^3^ Oxford Health NHS Foundation Trust UK

**Keywords:** appearance, body image, paranoia, persecutory delusions, self‐concept

## Abstract

**Objective:**

Persecutory delusions typically build on feelings of inter‐personal vulnerability linked to negative views of the self. Negative body image is an overlooked aspect of this link between the self‐concept and paranoia.

**Design:**

This study explores body image from the first‐person perspective of patients with persecutory delusions.

**Method:**

Semi‐structured interviews, analysed using interpretative phenomenological analysis, were conducted with twelve patients with persecutory delusions in the context of psychotic disorders.

**Results:**

Four super‐ordinate themes emerged. First, *appearance as a source of threat* accounted for how negative body image increased feelings of vulnerability and fed into the content of paranoia and voices (e.g., ‘I feel that everybody is noticing that I'm getting bigger and bigger and laughing at me’). Second, there was the negative *impact of uncontrollable and unwanted weight gain,* especially following antipsychotic medication (e.g., ‘I ballooned up to 23 stone’). Third, *feeling stuck* captured the hopelessness and resignation in relation to appearance (e.g., ‘I've become so accustomed to being overweight that I've accepted it as my lot’). Finally, *looking well symbolises feeling well* represented the importance of appearance in determining mental well‐being (e.g., ‘If I've got clean clothes and I put makeup on, at least I feel that I'm looking after myself’).

**Conclusions:**

Patients with persecutory delusions described appearance‐related concerns making them feel negative towards themselves, inferior to other people, and vulnerable to harm. Appearance‐related distress was broader than weight gain, including dissatisfaction with skin, clothing, and attractiveness. Negative body image may be a contributory factor in the occurrence of paranoia.

**Practitioner points:**

- Body image concerns may be of particular relevance in patients with persecutory delusions due to weight gain, inactivity, and medication side effects.
- Body image concerns include weight gain and broader aspects of appearance.
- Negative body image contributes to feelings of vulnerability, potentially worsening paranoid fears.
- It may be helpful for practitioners to explore the psychological impact of weight gain and body image concerns in patients with psychosis.

## Background

Body image forms one of the key components of an individual's self‐concept, that is, what a person thinks about one's self (Simons, Capio, Adriaenssens, Delbroek & Vandenbussche, 2012). It has been hypothesized that holding negative ideas about the self leads to feelings of being inferior and that paranoia builds upon this sense of vulnerability (Freeman, 2016). A number of systematic reviews conclude that negative self‐concept is a contributory causal factor in the development and persistence of persecutory delusions (Kesting & Lincoln, 2013; Tiernan, Tracey & Shannon, 2014). However, one potentially important component of self‐concept has been overlooked: body image.

Given the high rates of obesity in patients with psychosis, body image may be of particular relevance (Holt & Peveler, 2009). Significant weight gain is associated with low self‐esteem, social isolation, poor quality of life, and medication non‐compliance (McCloughen & Foster, 2011; Weiden, Mackell & McDonnell, 2004). Potential causes of obesity in patients include the adverse effects of antipsychotic medication, genetic vulnerabilities, metabolic syndrome, psychosocial risk factors, and unhealthy lifestyles (Bak, Fransen, Janssen, van Os & Drukker, 2014; Manu *et al.,* 2015; McEvoy *et al.,* 2005). Indeed, weight gain in itself is a side effect of antipsychotic medication (Allison *et al.,* 1999). Low mood, low self‐esteem, and social withdrawal resulting from excess weight may exacerbate persecutory fears. Another important consideration is the social context: both schizophrenia and obesity are associated with pervasive stigma (Carr and Friedman, 2005; Puhl and Heuer, 2009). Exposure to stigma regarding weight contributes to low self‐esteem and social isolation. This potential double stigma, of both excess weight and psychosis, may fuel social evaluative concerns upon which paranoid fears build.

Despite the plausible mechanistic link, there is almost no literature assessing body image and paranoia. In an analysis of two national epidemiological datasets, we found that negative body image was significantly associated with both mild and severe paranoia (Waite & Freeman, 2017). The association of body image and paranoia remained after controlling for body mass index (BMI) and gender. In a study of 167 patients with schizophrenia spectrum disorders (SSDs), a significant association between negative body image and low self‐esteem has been found (Oh, Sing and Shin, 2017). However, no study has investigated in patients with psychosis, the experience of body image and the potential links to paranoia or other psychotic experiences.

To date, there have been four qualitative studies exploring the experience of weight gain in patients with psychosis (Haracz, Hazelton & James, 2018; Usher, Park & Foster, 2013; Vandyk & Baker, 2012; Xiao, Baker & Oyewumi, 2012). All studies described rapid and uncontrollable weight gain following diagnosis and treatment, with one study describing the experience as a ‘double whammy’ (Haracz *et al.,* 2018). Patients emphasized the negative and widespread impact of weight gain on their physical, social, and psychological well‐being, often resulting in medication non‐adherence. These studies indicate the importance of weight gain as a clinical issue. However, none explored the experience of weight gain in relation to psychotic experiences nor considered the broader concept of body image or changes in appearance related to adverse effects of medication beyond weight gain (e.g., acne, hirsutism, cataracts, dry mouth; Seeman, 2011).

Body image concerns in patients with persecutory delusions have not received attention. Our first step is to explore body image from the patient perspective. Interpretative phenomenological analysis (IPA), with its focus on the subjective experience and the sense‐making process, will enable investigation of the nature and experience of body image concerns and how they may be understood from the first‐person perspective. The aim of this study was to build a greater understanding of body image in individuals with persecutory delusions. Potential gender differences will be explored.

## Method

### Design

This was an exploratory qualitative IPA study utilizing semi‐structured interviews to explore body image in patients with persecutory delusions. The study had full ethical approval from the NHS Health Research Authority (HRA) (ref. 17/SC/0530).

### Recruitment

In line with IPA methodology, a purposive homogeneous sampling technique identified potential participants with experience of persecutory delusions, for whom the research question was meaningful (Smith, Flowers & Larkin, 2009). The inclusion criteria were as follows: experience of persecutory delusions (current or historical) within the context of a primary diagnosis of a non‐affective psychotic disorder; aged 18‐65 years old; willing and able to provide informed consent; and sufficient command of the English language to participate in the interview. The exclusion criteria were as follows: diagnosis of moderate or severe learning disability; acute psychotic experiences that significantly affected functioning; current high risk of harm to self or others; a primary diagnosis of substance misuse or personality disorder; or significant forensic history. Potential participants were approached by a mental health professional involved in their care and were given a participant information sheet detailing the study aims and procedures. Of the 17 people approached, one declined, four did not meet the inclusion criteria, and twelve agreed to participate. Written informed consent, including consent to the use of pseudonymized quotes, was obtained.

### Materials

The interview schedule was developed through an iterative process which involved: consultation with a patient advisory group including service users and carers, review of the existing literature, and consideration of IPA methodology. The interview schedule explored five key topics: overall experience of body image, body image in day‐to‐day life, in relation to others, and in relation to psychotic experiences, and finally, the perspectives of others on appearance (Appendix [Link]). The schedule was used flexibly, and a range of prompts employed to stimulate discussion and respond to the narrative given by the participant.

### Procedure

Interviews were conducted by EM at participants’ homes or their local mental health clinic. Before the interview, participants were invited to choose a pseudonym for illustrative quotes. Interviews were audio‐recorded and transcribed verbatim. Interviews ranged in duration between 30 and 65 min (mean: 52 min).

### Analysis

Interpretative phenomenological analysis (Smith, 1996) was used to analyse participants’ accounts. IPA provides an idiographic framework for focusing on specific experiences by understanding how participants make sense of their experience, whilst simultaneously accounting for the role of the researcher in analysing and interpreting responses. The analysis (conducted by EM and supervised by FW & DF) combined IPA methodologies outlined by Smith et al. (2009) and Larkin & Thompson (2012).

Firstly, each interview was read and re‐read to increase familiarity. Next, *exploratory comments,* including descriptive, linguistic, and conceptual comments were noted. Links between *experiential claims* and *objects of concern* for the participant were recorded for each participant, alongside a reflection on the *tone and stance* of the interview. *Emergent transcript themes*, reflecting participants’ verbatim comments combined with interpretation by the analyst, were identified and labelled. Themes were then clustered and organized into case‐level *super‐ordinate themes* for each individual. Transcripts were analysed individually, then as a whole group by comparing and clustering *emergent transcript themes* to form *super‐ordinate* and *subordinate themes*. Convergence and divergence between the participants and within each individual account were considered, with nuances between genders highlighted.

The Yardley (2008) criteria for ensuring quality in qualitative research were used throughout the study, with specific adaption for IPA as informed by Smith et al. (2009). The criteria include sensitivity to context, commitment and rigour, transparency and coherence, and impact and importance. To ensure rigour, credibility checks were completed. A local network of IPA researchers independently analysed sections of transcript and an IPA expert provided feedback on the coding at case level and thematizing at group level. The principal researcher (EM) kept an audit trail of decision‐making. IPA acknowledges the inherent tensions of the researcher's potential influence over interpretation of the data (Smith *et al.,* 2009). Therefore, the principal researcher completed a reflexive log throughout the research process, to consider the potential influence on the research. Prior to data collection, a ‘bracketing’ interview was completed by EM, to aid identification of underlying assumptions and biases. It should be acknowledged that the research was conducted within a clinical research group interested in understanding and developing psychological treatments for distressing psychotic experiences. The researchers are all clinical psychologists. There were no prior relationships between the researcher conducting the interviews and the participants. All participants were aware that the research would contribute towards an academic qualification for the first author.

## Results

### Contextualizing the data

The characteristics of the participants are presented in Table 1.

**Table 1:** Participant characteristics

Pseudonym	Gender	Age	BMI	Age of onset of psychosis
Cathy	F	31	40.2	18
Echo	M	41	31.0	17
Hillary	F	47	28.7	24
John	M	48	41.7	35
Lin	F	42	27.1	37
Mandy	F	58	41.2	23
Melissa	F	40	35.6	12
Penelope	F	47	27.1	39
Percy	M	19	27.9	13
Robert	M	51	31.7	25
Sabastian	M	54	52.1	37
Yoda	M	37	24.2	36

The age of participants ranged from 19 to 58 with a mean age of 43 years (*SD* = 10.6). The mean BMI was similar for males (34.7, *SD* = 10.3) and females (33.3, *SD* = 6.5). One participant was in the healthy BMI category (18.5–24.9); four were in the overweight category (25.0–29.9); and seven were in the obese category (30.0+). Self‐reported age at the time of onset of psychotic experiences ranged from 12 to 39 with a mean age of 26.3 years (*SD* = 10.6).

Participants had a range of clinical diagnoses including paranoid schizophrenia (n=7), schizoaffective disorder (n=4), and psychosis not otherwise specified (n=1). Eleven participants were taking antipsychotic medication and five had recently completed a psychological intervention. One participant (Yoda) was recruited from an early intervention service having been recently diagnosed and had not yet received any psychological or pharmacological treatment.

At the time of recruitment, nine participants were single and three participants were married. Six participants lived alone, three lived with their partner, two were living with their family of origin, and one lived in supported housing. No participants were currently in paid employment. Four participants were engaged in voluntary work, and one was a full‐time unpaid carer. Eleven participants were white British and one participant was Chinese.

The analysis resulted in four super‐ordinate themes, each with corresponding subordinate themes (represented in italics) that capture the shared and contrasting experiences of the participants. Table 2 represents the overall structure and recurrence of the super‐ordinate and subordinate themes.

**Table 2:** Structure and recurrence of super‐ordinate and subordinate themes

Super‐ordinate and subordinate themes	Total	Cathy	Echo	Hillary	John	Lin	Mandy	Melissa	Penelope	Percy	Robert	Sabastian	Yoda
**1. Appearance as a source threat**	12	–	–	–	–	–	–	–	–	–	–	–	–
(1a) Humiliated by my appearance: ‘people don't want to sit next to me’ (Penelope)	11	–	–	–	–	–	–	–	–	–		–	–
(1b) Body image concerns increase mental health difficulties: ‘they're talking about me…they're judging me from how I look’ (Percy)	10	–	–	–	–	–	–		–	–	–	–	
(1c) Desperation to ‘fit in’: ‘I want to be accepted in society. I want to feel like I belong’ (Hillary & Penelope)	7			–		–	–		–	–		–	–
**2. Impact of uncontrollable and unwanted weight gain**	11	–	–	–	–	–	–	–	–	–		–	–
(2a) Devastation of weight gain: ‘I'm covered in scars’ (Echo)	7		–	–	–	–		–	–	–			
(2b) Medication‐induced weight gain: ‘I got fat’ but ‘it's not my fault’ (Lin & Melissa)	3		–			–		–					
(2c) Medication as a double‐edged sword: ‘It keeps us out of hospital’ (Melissa)	6	–		–			–	–	–	–			
(2d) Weight gain is the problem but weight loss is not the full solution: ‘Even when I lost a load of weight… I just don't like myself’ (Mandy).	8	–	–	–	–		–			–		–	–
**3. Feeling stuck**	12	–	–	–	–	–	–	–	–	–	–	–	–
(3a) Resignation and hopelessness: ‘I can't do much about how I physically look’ (Percy)	10	–	–	–	–	–	–	–	–	–		–	
(3b) Perseverance and coping: ‘I tell myself to stop worrying’ (Cathy)	10	–	–	–	–	–	–	–	–	–	–		
(3c) Attempting to regain control: ‘My body does things that I don't want it to do’ (Hillary)	10	–	–	–	–	–	–	–	–	–	–		
(3d) Pain of confronting difficulties: ‘I don't really want to go into it’ (Echo)	5	–	–	–				–		–			
(3e) Aiming towards self‐acceptance: ‘I have to start valuing myself’ (Penelope)	7	–		–				–	–	–	–		–
**4. Looking well symbolises feeling well**	10	–		–			–	–	–	–	–	–	–
(4a) Keeping up appearances: ‘if I've got clean clothes and I put makeup on, at least I feel that I'm looking after myself’ (Hillary)	10	–		–			–	–	–	–	–	–	–
(4b) Acceptance by others boosts confidence: ‘I realise that I am wanted’. (Melissa)	5	–					–	–	–			–	

### 1 Appearance as a source of threat

The first super‐ordinate theme encapsulates the role of appearance‐related distress in feeling humiliated, rejected, and vulnerable to harm. Eleven participants described the experience of being *(1a) Humiliated by my appearance: ‘people don't want to sit next to me’ (Penelope)*. Emotive accounts reflected the felt sense of being judged and humiliated following weight gain: ‘someone called me a lump once, “what are you gonna do, you fat lump?”’ (Echo, 454) and ‘I start sweating and everyone will look at me and say, “Eugh, you're really messy”’ (Hillary, 8). Participants reflected on feeling ashamed of their physical appearance: ‘My friends have commented saying my teeth are black’ (Lin, 312) and ‘when I used to have a shaved head, he used to say “you look like a thug”’ (Echo, 199). For some, this sense of humiliation was rooted in painful childhood experiences:

I was brought up by a step‐dad, and he basically said to me every day, ‘you're ugly you are, you'll never get a wife.’ (John, 13)

My mum and dad didn't have much money, I had some jumble sale stuff sometimes and it was embarrassing. And that triggered me off going down the street with people looking at me. (Mandy, 244)

Participants were acutely aware of being ostracized by society based on judgements regarding their physical appearance, explaining that ‘they treat you with absolute contempt (…) I almost feel like I have to apologise for myself’ (Penelope, 287). In her account, Penelope articulated the direct impact of being dissatisfied with her appearance and this contributing to feelings of isolation:

But now, virtually nobody talks to me. I don't know if that's my body language. I think maybe it's because I'm avoiding eye contact and sort of keeping myself to myself and I do it subconsciously (…) or whether it's because I've got a scary face (361)

This account highlights the perception that individuals in society prioritize physical appearance above personality traits: ‘I'm much nicer, but I've put on weight and (…) I feel a lot of people don't want to talk to me’ (Penelope, 369). Body image concerns led to a number of avoidant coping strategies: ‘I withdraw if there's a possibility to withdraw’ (Penelope, 43), ‘I don't want to see anybody and I'm ashamed to look people in the face’ (Hillary, 335), and ‘I don't like smiling at people and stuff like that because of my teeth’ (Echo, 22).

Further to societal alienation, participants’ accounts emphasized feeling unworthy of love, care, and friendship in personal relationships, resulting from dissatisfaction with their physical appearance: ‘I'd think *why would they* want to be friends with me if I'm fat, kind of thing, and ugly?’ (Percy, 144) and ‘I just think, no‐one would want me anyway’ (Echo, 211). For some, this felt very certain and unchangeable, ‘people don't like me because I'm not very beautiful’ (Cathy, 126) and for others being liked was predicated on losing weight, ‘I think if I lost some weight, they'd like me more’ (John, 32). In one account, Echo highlighted the relationship between his dissatisfaction with his physical appearance and how others perceive him: ‘I'm just not happy with the way I am, what I look like, what I look like to other people’ (190).

Most participants suggested that *(1b) Body image concerns increase mental health difficulties: ‘they're talking about me…they're judging me from how I look’ (Percy)*. This subordinate theme captured the direct impact of negative body image on the exacerbation of psychotic experiences, self‐harm and suicidality, low mood, and negative self‐concept. Participant accounts reflected the poignancy of experiencing an extreme aversion towards one's own appearance and the detrimental impact this had on their mental health.

Participants linked their negative body image to increased paranoid ideation, with five participants speaking directly about the interaction with their psychotic experiences (i.e., hearing voices or experiencing persecutory delusions). These participants described how ‘harassed’ (Lin, 259), ‘vulnerable’ (Echo, 63), and ‘exposed’ (Percy, 178) they felt when their voices and paranoid ideation focused on their physical appearance. Echo gave an evocative account of the exploitative nature of his voices:

It's because of the voices, I can hear them (…) they just make comments on what I look like (…) they know all your fears and everything like that and all your insecurities and they just play on it. (66)

Participants also described how their voices ‘pick faults’ (Echo, 271) and ‘can be really cruel’ (Melissa, 80) about their appearance. Mandy gave an example of how negative body image contributes to her negative self‐concept: ‘I just think I'm ugly. It's the way people look at me sometimes and I think they're thinking “she's ugly‐ why is she so ugly?”’ (293). Mandy further highlighted how the content of her voices contributes to her negative self‐concept leading to increased ideas of reference: ‘You are ugly, you don't deserve nobody (…) you're ugly, people are looking at you, look at them looking at you’ (308). Together, these accounts emphasize an experience of appearance‐related concerns in the context of a negative self‐concept that, for this person, appeared to increase their vulnerability to distressing voices and paranoid ideation.

Participants spoke about heightened fears about others’ reactions to their appearance: ‘I feel that everybody is noticing that I'm getting bigger and bigger and laughing at me’ (Mandy, 356), ‘you can tell that they're talking about me and I'm just very mindful of the fact that they're judging me from how I look’ (Percy, 169). Concerns about appearance were an important component of paranoid fears and ideas of reference:

Those times are hard because people are physically there to judge, and criticise. And even if they don't physically say stuff, I'll worry that they could be. They might not say ‘you're fat’ but I might be worried that they might think I'm fat or ugly. (Percy, 112)

For some, body image was directly linked to persecutory ideation: feeling vulnerable and under threat from others as a result of physical appearance:

It's the people walking past me or behind me. I have a fear of men walking behind me. I feel they are laughing at me and, you know, and they're gonna get to me and it's horrible. *I: Why do you think they might be laughing at you?* I don't know, it's the way I probably look. (Mandy, 74)

Ideas of persecution extended beyond weight concerns and general appearance to include people's clothing choice: ‘I had an artificial fur coat (…) this young lad went past me and I felt he bumped into me on purpose and said “you dog”’ (Mandy, 86). In contrast, Melissa described an awareness of the unfounded nature of her fears about others: that despite her own body image concerns and fears about others’ reactions, appearance was not necessarily a source of criticism or hostility from others:

I know I'm big but it's not like people shout at me in the street or something like, ‘oh fatty.’ It's not like that, kids shouting or anything. They don't, that's the thing, they don't. (311)

Three participants described how negative body image led to distressing episodes of deliberate self‐harm or suicide ideation: ‘I cut my face ‘cos I wanted to look different, I cut my face with a razor’ (Echo, 17). For some, battling with weight gain resulted in feeling ‘sad and miserable and quite lonely’ (Percy, 188), with an internalized sense of failure:

I'm depressed most of the time. I'm angry with myself that I'm overweight. I don't like being overweight. And every time I go to Slimming World, and I haven't lost very much, I get angry with myself. (John, 51)

Most participants expressed dissatisfaction with ‘the way I am, the way I look’ (Echo, 30), highlighting that poor body image is a component of self‐concept. Participants described how changes in their appearance impacted their self‐concept: ‘made me lose confidence in myself’ (Echo, 415), ‘I've kind of lost self‐respect. I used to try really hard to look after myself’ (Penelope, 57).

Seven participants emphasized a longing to be accepted by others in the subordinate theme, *(1c) Desperation to ‘fit in’: ‘I want to be accepted in society. I want to feel like I belong’ (Hillary & Penelope)*. This theme encompassed participants’ feelings towards their appearance and mental health. Participants described feeling like an ‘outsider’ (Penelope, 294), that the way they dress is ‘wrong’ (Hillary, 162), and feeling as though ‘I've got to do this stuff to fit in’ (Hillary, 151), for example, dressing and behaving in a particular way. The accounts highlighted the sense of exclusion with remarks such as ‘why can't I be normal?’ (Mandy, 457).

For a number of patients, body image concerns became more pertinent as they sought to move on after recovering from an acute psychotic episode: ‘when you're in hell, what you look like doesn't matter’ (Lin, 57), but ‘body image has become more of a problem now I am trying to get more in the real world’ (Percy, 262) as now, ‘I just care more about what people think about how I look’ (Percy, 65).

There were very few gender differences within this super‐ordinate theme. One notable difference was that males tended to talk more emotively about wanting to feel accepted within relationships with friends and family, whereas female participants spoke more about wanting to be accepted within general society, with a strong desire to ‘fit in’, ‘belong’, and be ‘normal’.

### 2 Impact of uncontrollable and unwanted weight gain

All but one of the participants described their experiences of weight gain and the highly negative impact this had on their relationship with themselves and others. (*2a) Devastation of weight gain: ‘I'm covered in scars’ (Echo)* encapsulates the experience of rapid weight gain after starting antipsychotic medication: ‘I ballooned up to 23 stone’ (Echo, 24) and ‘I couldn't recognize myself hardly’ (Hillary, 138). Particularly for Echo, drastic measures were taken to try to reverse the impact of weight gain: ‘most of my tattoos were to cover up the scars’ (96), ‘I took amphetamines for a few years to lose weight’ (296), and ‘I didn't eat for days sometimes’ (310). Evocative accounts highlight the experience of losing control over appetite following a psychotic episode:

Becoming ill with schizoaffective disorder was so dramatic and happened so unexpectedly, I became psychotic so quickly, I started eating vast amounts of food (…) I went from eating virtually nothing to gorging on packets of crisps, and chocolate bars, and fish and chips, and everything that you could think of that was unhealthy. (Penelope, 193)

Three participants noted that weight gain was a side effect of medication and therefore not their fault, in the subordinate theme, *(2b) Medication‐induced weight gain: ‘I got fat’ but ‘it's not my fault’ (Lin & Melissa)*. One participant explained, ‘it's not all your fault. It's medication. I'm on a lot of medication’ (Melissa, 4). This gave some a sense of devolved responsibility, for example, knowing ‘you can't help it (…) makes me feel a bit better’ (Melissa, 328). Others highlighted the lasting impact of weight gain after stopping medication: ‘They took me off it [medication] after a year or two, but I'd already put on the weight then’ (Echo, 143).

The importance, as well as unwanted effects, of medication was recognized in the subordinate theme *(2c) Medication as a double‐edged sword: ‘It keeps us out of hospital’ (Melissa)*. Participants gave striking accounts highlighting the ‘constant battle’ (Percy, 132) between weight management and symptom management on antipsychotic medication:

Yeah, I think it's hard with the medications because you're trying really hard to diet but then your medication is making you crave food and you're sort of in a constant battle to try and diet and you just can't do it because the medication's making you crave the food. (Percy, 132)

Well, obviously everyone would not want to be on medication, but if it keeps us well, it keeps us out of hospital, it's just something that people have to accept. (Melissa, 198)

A vicious cycle of weight gain, low mood, and eating to manage emotions was identified:

You put on weight and then, for me I get disheartened when I put on weight and then I tend to eat more because I'm upset that I've put on the weight, and it's kind of a bit of a downward spiral. (Percy, 134)

Some participants had successfully managed to lose weight, yet this did not improve self‐concept: *(2d) Weight gain is the problem but weight loss is not the full solution: ‘Even when I lost a load of weight… I just don't like myself’ (Mandy)*. Participants explained ‘I don't really feel better for losing the weight. It just feels that I've lost it in all the wrong places’ (Hillary, 267) and ‘I still wasn't happy with myself’ (Echo, 176). Sabastian described ‘my body changed outside but my mind hadn't’ (93). Initially, he described feeling ‘over the moon’ (87) but later acknowledged ‘I couldn't deal with what was going on’ (95). This account illustrates an unforeseen, catastrophic impact of losing weight, resulting in unwanted expectations about change that pre‐empted a relapse of psychotic symptoms. Some participants emphasized that changes in their appearance impacted their sense of self and interactions with others. The only patient who had not commenced pharmacological treatment and whose BMI was not in the overweight or obese category reported no issues with weight: ‘my weight is not really that much of a problem with me’ (Yoda, 182).

### 3 Feeling stuck

This super‐ordinate theme captured the overwhelming sense of hopelessness, resignation, and frustration with regard to appearance. Ten participants described *(3a) Resignation & hopelessness: ‘I can't do much about how I physically look’ (Percy)*. The accounts highlighted the challenge of living with changes in appearance and the loss of hope and sense of defeat:

I need to change, and stop bloody dwelling on it all and do something about it, but I don't quite know where to start. (Hillary, 413)

I've become so accustomed to being overweight that I've accepted it as my lot. I've almost given up hope of ever being slim again. (Penelope, 177)

I just (sigh), accept it, try and accept it, you have to, otherwise you're just going to make yourself angry the whole time. (Echo, 258)

The subordinate theme, *(3b) Perseverance and coping: ‘I tell myself to stop worrying’ (Cathy)*, encapsulates unrelenting perseverance and the pursuit of finding ways to cope. Some participants emphasized their commitment to weight loss, such as ‘I always try and do at least an hour walk a day’ (Percy, 124), whilst others talked about their attempts to avoid worrying about their appearance, for example, ‘I try not to worry about it too much, you know, try and sort of get on with my life and try to enjoy it the best that I can’ (Melissa, 158). For some participants, perseverance reflected hope : ‘I think if I worked on my appearance a bit more, it might help me to feel better about myself’ (Cathy, 229), whereas others felt dependent on others for support: ‘If people would come with me, I'd get the exercise’ (John, 313).

Ten participants described an ongoing battle characterized by a loss of agency and control in the subordinate theme, *(3c) Attempts to regain control: ‘My body does things that I don't want it to do’ (Hillary)*. Participants described feeling at a loss and expressed fluctuating levels of hope and despair:

I just feel lost with it, my weight. I have a splitz of inspiration and hope and I think, ‘Oh, I'm going to do it this time.’ Then I just end up eating again. It's really frustrating. (Penelope, 179)

One participant reflected on ways of maintaining control of her eating by setting firm rules: ‘try and be strict with yourself and not overdo it or overindulge’ (Melissa, 300).

Five participants acknowledged the ‘uncomfortable’ (Percy, 299) and ‘awkward’ (Melissa, 296) nature of discussing their ‘personal’ (Echo, 504) experience of their body image in the theme *(3d) Pain of confronting difficulties: ‘I don't really want to go into it’ (Echo)*. Linguistically, this was characterized by hesitations, tangential responses, and tailing off, but was also more directly addressed in participant accounts: ‘I don't really want to talk about that’ (Cathy, 151). For Echo, it was clear that this had not been talked about before and was linked to feeling vulnerable:

I don't wanna go there, I don't really want to go into it. (…) It's difficult. It's hard to talk about, I haven't really told, I don't really tell anyone about things like that’. (88)

In contrast, Hillary described feeling regret at not speaking more openly about her appearance earlier on in her life and not asking for help, saying that ‘I would have talked to someone and explained how I felt about my body’ (426).

Seven participants highlighted developing a realization of the importance of *(3e) Aiming towards self‐acceptance: ‘I have to start valuing myself’ (Penelope),* linking this to social acceptance: ‘Maybe, if I liked myself, it wouldn't matter if other people didn't like me’ (Cathy, 201). For others, self‐acceptance began with finding forgiveness for the self: ‘I have to forgive myself because that was then and this is now. I turned out to be a really nice woman’ (Melissa, 74) and self‐compassion: ‘there's that little person inside of me that's not nasty at all, you know. Scared and lots of feelings going on but not horrible’ (Hillary, 317). For Yoda, being true to one's self was an important theme: ‘How I perceive myself is more important than how other people see me’ (42). Similarly for Robert, a better understanding of himself was key in taking back control of his mind from a position of self‐acceptance: ‘When you have understanding of yourself, you have understanding of what you are and what you can do about what's going on’ (469).

### 4 Looking well symbolises feeling well

The final super‐ordinate theme captures the importance of maintaining physical appearance as an indicator of mental wellness to oneself and others. Ten participants described the experience of *(4a) Keeping up appearances: ‘if I've got clean clothes and I put makeup on, at least I feel that I'm looking after myself’ (Hillary)*. Self‐care was particularly important for the female participants, examples including, ‘It's important to look after yourself, have a shower, put a bit of makeup on’ (Melissa, 425), ‘I try and put clean clothes on, I try and do my hair. I try and do my teeth’ (Hillary, 329), and ‘I keep myself clean and I try to wear clean clothes’ (Penelope, 237). For four females, this also extended to the appearance of their home: ‘My house is not very tidy (…) I guess I don't want to invite people to my place’ (Lin, 108).

This emphasis on self‐care and appearance of the home environment was less prominent in the male accounts; however, there was an acknowledgment that ‘you have more good feelings about yourself when you look better’ (Robert, 185). Both males and females felt that their clothing choice was important for boosting their confidence and portraying wellness to others, for example, ‘I try to dress smart, present as respectable’ (Percy, 73) and ‘I'm not indecent, nothing's falling down (…) I do try and wear clothes that are appropriate’ (Melissa, 216). For some, clothing choice was directly linked to a fear of judgement from others:

I don't want to be that negative, black hole, standing on other people's energy and happiness, or whatever. (Lin, 90)

For some participants, recovery from psychosis was judged by how well they look, for example, ‘If I'm like not well or well to other people, they can read what I'm like’ (Robert, 9). Conversely, for others, physical appearance made little difference to their sense of recovery: ‘It doesn't matter if you put makeup on or wear your clothes, clean clothes, you're still fucked up’ (Hilary, 360). Reassuring self‐talk was one potential strategy for helping to feel reintegrated in society, for instance, ‘I'm constantly thinking right, “Just do the best you can and then people will be nicer to you,” and err “You won't look as weird as you have done”’ (Penelope, 250).

Four female participants reflected on what helps them feel better about themselves and their appearance, in the subordinate theme *(4b) Acceptance by others boosts confidence: ‘I realise that I am wanted’. (Melissa)*. For Melissa, being respected by others and being complimented helped her build confidence: ‘It boosts me up and makes me feel better when I'm paid compliments by other people, that really helps’ (420), whereas having a loving relationship helped Lin: ‘I feel I'm in a secured situation’ (48). Being accepted by non‐judgmental others was important for self‐acceptance: ‘It's not like my friends judge me or anything and I don't judge them’ (Melissa, 359). However, having a negative self‐concept may prevent feeling accepted by society despite positive comments or interactions with others, for example, ‘People can say you are a nice‐looking girl but I won't have it to myself. I don't know why. Perhaps I don't feel worthy’ (Mandy, 140).

## Discussion

This study explored the lived experiences of body image in twelve patients with persecutory delusions. Six males and six females participated, all of whom had experience of persistent persecutory delusions and were accessing clinical services at the point of recruitment. It is the first study to explore the possible link between body image and psychotic experiences, extending beyond the impact of weight gain due to antipsychotic medication. The topic produced rich accounts of the impact of body image on daily life.

The accounts provided insight into the link between body image concerns and psychotic experiences. Patients described negative experiences of their body image as a potential route to paranoia. Feeling negative about their appearance led to them feeling bad about themselves, inferior to those around them, and thus feeling vulnerable to harm from others. This fits with our existing understanding that negative self‐concept leads to feelings of inferiority, and hence vulnerability, conditions in which paranoia is hypothesized to thrive (Freeman *et al.,* 2014; Freeman, 2016; Collett, Pugh, Waite & Freeman, 2016). As such, body image concerns may contribute towards negatively held ideas about the self (i.e., negative self‐concept), which is an identified causal factor in the development and persistence of persecutory delusions (Freeman,^2016^). Patient accounts were consistent with the notion of a hierarchy of paranoia (Freeman *et al.,* 2005): many described their body image concerns in relation to ideas of reference (i.e., being observed, followed, discussed), with fewer accounts linking body image directly to persecutory ideation. Furthermore, participants identified appearance‐related concerns as featuring in their experience of critical and derogatory voices.

The experience of weight gain as rapid and uncontrollable was consistent with the findings reported in previous qualitative research (Haracz *et al.,* 2018; Usher *et al.,* 2013; Vandyk & Baker, 2012; Xiao *et al.,* 2012). However, in this study, participants emphasized a persistent ‘stuckness’. Despite efforts to persevere with weight loss, participants spoke about having very little control over their appearance, resulting in a sense of hopelessness and resignation. Consequently, body image concerns were an identified source of vulnerability to further mental health problems, such as depression, self‐harm and suicidality, as well as relationship and employment difficulties.

Patients with psychosis report significant barriers to their participation in exercise and weight loss interventions (e.g., Firth *et al.,* 2016). One potential explanation is that existing interventions have failed to recognize the relationship between body image concerns on other mental health problems. Importantly, participants who succeeded in weight loss continued to describe negative beliefs about themselves, and for one patient, weight loss may have contributed towards relapse. Therefore, further research is needed to investigate the psychological factors involved in interventions to target weight loss in patients with psychosis.

Body image concerns were broader than excess weight, encompassing dissatisfaction with facial features, skin, clothing, and physical attractiveness. Current practice within mental health services focusses predominantly on physical health monitoring and therefore potentially overlooking the impact of broader appearance‐related distress. Furthermore, participants emphasized the importance of appearance as an indicator of mental wellness and recovery both to oneself and others. Participant accounts indicated that by attending to their physical appearance, improvements in their mood and self‐concept were observed, leading to greater acceptance by others, and an increased sense of security and confidence. Efforts to improve the self‐concept of patients with persecutory delusions (Freeman et al., 2014) have not systematically addressed body image. Our study indicates that this may perhaps be a significant omission.

### Study limitations

There are a number of limitations to this study. One factor overlooked in the purposive recruitment of a homogeneous sample was the amount of time elapsed since diagnosis. Stage of recovery could potentially be an important consideration in differentiating the experience of body image. Secondly, the patients recruited in this study were an outpatient population from a single geographical area, all of whom had experienced persistent persecutory delusions and 5 of whom had received psychological input. All but one of the participants are white British; however, this reflects the population accessing these particular clinical services. As this study focused on understanding subjective experiences from the patient's perspective, it was not possible, nor the aim, to determine whether the accounts of persecution and threat were unfounded. This study was conducted by a research group focused on understanding the mechanisms which underpin psychotic experiences in order to develop effective psychological treatments. The most likely impact of this is greater focus on psychological mechanisms over biological or social. However, the aim of this study, in line with IPA research, was to use first‐person accounts to inform theoretical and clinical developments. We believe that the accounts described in this paper provide a resource to achieve this aim.

A programme of research that investigates the interaction between body image concerns, physical health, and mental health in patients with psychosis is needed.

## Funding

EM was supported by the Oxford Institute of Clinical Psychology Training Doctoral Course. DF holds an NIHR Research Professorship (NIHR‐RP‐2014‐05‐003).

## Supporting information


**Appendix S1.** Semi‐structured interview schedule.
