# Supplementary material for: The experience of body image concerns in patients with persecutory delusions: ‘People don't want to sit next to me’
Source: Psychol Psychother. 2019 Aug 10;93(3):639–55. doi: 10.1111/papt.12246 (PMC7496653; doi:10.1111/papt.12246)
Supplement: Supplementary file 1 — Appendix S1. Semi‐structured interview schedule. [file PAPT-93-639-s001.pdf]

Supporting information
Click here to access/download;Supporting information;Interview_schedule.docx

Semi-structured interview schedule

1. What do you understand by the term body image? What meaning does it hold for you?
2. What is your experience of your body image/appearance?
3. What has been your experience of your body image in day-to-day life?
4. Is there any link between your body image and relationships with others?
5. What ideas do you have about the way you think other people see your body?
6. Is there anything we have not asked about that you think might be important, or might help us to better understand your experiences?
